# What do primary care providers want to know when caring for patients living with frailty? An analysis of eConsult communications between primary care providers and specialists

**DOI:** 10.1186/s12913-024-10542-x

**Published:** 2024-01-16

**Authors:** Sathya Karunananthan, Giovanni Bonacci, Celeste Fung, Allen Huang, Benoit Robert, Tess McCutcheon, Deanne Houghton, Ramtin Hakimjavadi, Erin Keely, Clare Liddy

**Affiliations:** 1 Interdisciplinary School of Health Sciences, University of Ottawa, 200 Lees Ave #516F, Ottawa, ON Canada; 2 C.T. Lamont Primary Health Care Research Centre, Bruyère Research Institute, Ottawa, ON Canada; 3 Family First Health Centre, Ottawa, ON Canada; 4 Department of Family Medicine, University of Ottawa, Ottawa, ON Canada; 5 St Patrick’s Home of Ottawa, Ottawa, ON Canada; 6 Present Address: Ontario eConsult Centre of Excellence, The Ottawa Hospital, Ottawa, ON Canada; 7 Ottawa Hospital Research Institute, Ottawa, ON Canada; 8 Department of Medicine, University of Ottawa, Ottawa, ON Canada; 9 Perley Health Centre of Excellence in Frailty-Informed Care™, Ottawa, ON Canada; 10 Faculty of Medicine, University of Ottawa, Ottawa, ON Canada

**Keywords:** Frailty, eConsult, Primary care, Telemedicine, Specialist care

## Abstract

**Background:**

Frailty is a complex condition that primary care providers (PCPs) are managing in increasing numbers, yet there is no clear guidance or training for frailty care.

**Objectives:**

The present study examined eConsult questions PCPs asked specialists about patients with frailty, the specialists’ responses, and the impact of eConsult on the care of these patients.

**Design:**

Cross-sectional observational study.

**Setting:**

ChamplainBASE™ eConsult located in Eastern Ontario, Canada.

**Participants:**

Sixty one eConsult cases closed by PCPs in 2019 that use the terms “frail” or “frailty” to describe patients 65 years of age or older.

**Measurements:**

The Taxonomy of Generic Clinical Questions (TGCQ) was used to classify PCP questions and the International Classification for Primary Care 3 (ICPC-3) was used to classify the clinical content of each eConsult. The impact of eConsult on patient care was measured by PCP responses to a mandatory survey.

**Results:**

PCPs most frequently directed their questions to cardiology (*n* = 7; 11%), gastroenterology (*n* = 7; 11%), and endocrinology (*n* = 6; 10%). Specialist answers most often pertained to medications (*n* = 63, 46%), recommendations for clinical investigation (*n* = 24, 17%), and diagnoses (*n* = 22, 16%). Specialist responses resulted in PCPs avoiding referral in 57% (*n* = 35) of cases whereas referrals were still required in 15% (*n* = 9) of cases. Specialists responded to eConsults in a median 1.11 days (IQR = 0.3–4.7), and 95% (*n* = 58) of cases received a response within 7 days. Specialists recorded a median of 15 min to respond (IQR = 10–20), with a median cost of $50.00 CAD (IQR = 33.33 – 66.66) per eConsult.

**Conclusions:**

Through the analysis of questions and responses submitted to eConsult, this study provides novel information on PCP knowledge gaps and approaches to care for patients living with frailty. Furthermore, these analyses provide evidence that eConsult is a feasible and valuable tool for improving care for patients with frailty in primary care settings.

**Supplementary Information:**

The online version contains supplementary material available at 10.1186/s12913-024-10542-x.

## Background

Frailty often involves multiple chronic diseases, functional limitations, polypharmacy, and challenging social circumstances requiring access to various specialists, allied health professionals, and community services [1, 2]. Delays in access result in increased emergency room visits or hospitalizations where these patients often experience adverse outcomes and a precipitous decline in health [3, 4]. In order to effectively manage people living with frailty and the associated health care costs while ensuring the sustainability of health and social care settings, we need innovative models that promote access to elder-friendly care protocols and appropriate training for care providers [5]. Primary care providers (PCPs) are the first point of contact into the health care system for older adults and are increasingly being called upon to care for the growing number of those living with frailty [6, 7], yet there is still no clear training or guidance for frailty care and its identification and management remain complex and challenging [6, 8, 9].

To date, little is known about PCP approaches to frailty care and their knowledge gaps. Providers have reported a lack of training in frailty, of appropriate screening tools, and of clear pathways for frailty management, all of which may be hindering optimal care for patients living with frailty [9–11]. A better understanding of PCP knowledge gaps related to frailty based on the questions that arise when they encounter patients with frailty in their clinical practice can contribute to the development of educational activities tailored to their learning needs in order to improve the care and health outcomes of older adults [8, 11].

eConsult is a secure web-based tool that allows PCPs to submit questions about patient care and receive responses from specialists asynchronously [12]. Advice received through eConsult often allows PCPs to tailor a patient’s care without the need for a face-to-face specialist visit [13]. eConsult communication logs between PCPs and specialists are a unique source of data that can be studied to gain insights on questions PCPs ask about specific patient populations.

In this study, we analyze eConsult data to describe the types of questions PCPs ask about patients they identify as “frail”, corresponding specialist responses, and the impact eConsult has on the care of these patients. Through our analysis, we address the following questions: What types of questions and what clinical topics do PCPs ask on eConsult when caring for older adults identified as “frail”? To which speciality groups are PCPs directing their questions? What types of responses do specialists provide to PCPs through eConsult when responding about patients with frailty? What is the impact of the eConsult on the course of action and need for referrals for older patients with frailty? What impact does eConsult have on care and access to services for patients with frailty?

## Methods

### Study setting

The ChamplainBASE™ eConsult service is a secure web-based platform that allows a PCP to submit non-urgent clinical questions to specialists from over 140 specialty groups offered through the platform. To submit a question, the PCP accesses the platform through a web browser, enters the question accompanied by the relevant patient information and history, and sends the case to a selected specialty group. PCPs can attach supplemental information including imaging reports, laboratory results, or multimedia files (pictures or videos). An email is then sent to a specialist who will review the eConsult and either provide a recommendation for management, request more information, or recommend an in-person referral. Once the PCP receives the specialist’s recommendation and has no follow-up questions, the eConsult is completed and the PCP fills out a mandatory five-question survey with the option to include comments.

### Study design: case level data

This is a cross-sectional study of frailty-related eConsult cases closed between January 1, 2019 and December 31, 2019. Case inclusion was based on the presence of the word “frail” or “frailty” in the case details text and patients who were 65 years or older.

### eConsult utilization data

The following utilization data are routinely collected through the eConsult system: patient age and sex, PCP type (physician or nurse practitioner), PCP and specialist geographic regions, specialty groups consulted, time spent by the specialist in generating a response, cost of specialist consultation, and number of exchanges between the PCP and specialist.

### Classifications of eConsult questions and responses

To classify eConsult questions related to frailty, we adapted two previously validated taxonomies. The Taxonomy of Generic Clinical Questions (TGCQ) was used to classify PCP questions into eight broad categories: diagnosis, management, drug treatment, non-drug treatment, epidemiology, education, nonclinical, and unclassified [14]. Each category included multiple subcategories. For example, drug treatment included how/when to prescribe, how/when to deprescribe, safety/adverse effects/interactions, and other. See Appendix A for a complete list of the categories considered. For eConsult cases that included multiple questions, each question was classified separately.

The International Classification for Primary Care 3 (ICPC-3) was used to classify the clinical content of each eConsult [15]. Sixteen relevant categories were considered (e.g., respiratory system, urinary system, social problems, functioning) and for each, sub-categories were also included (e.g., psychological system: delirium, memory or attention problem, depressive disorder). See Appendix B for the complete list of categories considered.

The TGCQ and ICPC-3 taxonomies were adapted to this study by team members with expertise in frailty (SK & MHS) and four clinician coders with expertise in caring for older adults (GB, CF, AH, BR) to include categories that would be relevant to patients living with frailty.

Specialist responses were classified into one of eight types using a taxonomy previously developed for ChamplainBASE™ eConsult analyses: diagnosis, screening recommendation, investigation recommendation, medications (start/stop/rationale), medications (monitoring/complications), non-pharmacological therapy, complications/comorbidities, and other.

In addition, specialist recommendations regarding referral were coded for whether the case required no referral, a potential future referral, immediate referral to a specialist, to specialized geriatric assessment, to an allied health professional, or to a non-specialized community service. Finally, coders noted whether specialists referenced specific guidelines or sources of information in their response.

Four clinicians (GB, CF, AH, BR) were responsible for coding cases. All four clinicians first coded the same twenty cases to validate the taxonomy. Following an analysis of discrepancies in the coding and a few modifications to the taxonomy for additional clarity, the remaining cases were coded by two of the four coders and consensus was achieved through discussion.

### Impact of eConsult on care for patients living with frailty

Before an eConsult case is closed, the PCP must complete a mandatory five-question close-out survey. Questions pertaining to the impact of the eConsult on PCP’s course of action, decision to refer, and PCP satisfaction were included in the current analysis (See Appendix C for survey questions).

### Study analysis

We conducted descriptive analyses on the utilization data, classification of PCP questions and specialist responses, and PCP responses to the close-out survey. We provide frequencies to summarize PCP question types and content, specialist referral recommendations, and resources cited. Summary statistics including mean (standard deviations), median (interquartile range) and frequency were used to describe utilization data including patient age and sex, response iterations and time, specialty distribution, specialist time billed per eConsult, and cost of an eConsult.

## Results

### eConsult utilization data

Among the 16,766 eConsult cases closed between January 1, 2019 and December 31, 2019, we identified 76 that included the term “frail” or “frailty” and involved patients over 65 years of age. Of these, 61 were included in the current study. The remaining 15 cases were excluded because the term ‘frail’ was not used to describe the patient. For example, the PCP may have mentioned that the patient is “not frail” or referred to the patient’s caregiver as frail.

eConsult utilization for cases related to frailty are summarized in Table 1. The age of patients included ranged between 66.0 and 101.2 years, with a mean of 85.7 years (SD = 8.79) and 75% (*n* = 46) of the eConsult cases were for female patients. Of the 61 eConsult cases, 87% (*n* = 53) were submitted by physicians and the remaining 13% (*n* = 8) by nurse practitioners. PCPs associated with long-term care settings sent 11% (*n* = 7) of the eConsults. PCPs in the Champlain region sent 89% (*n* = 54) of the eConsults and specialists in the Champlain region responded to 90% (*n* = 55) of them. The remaining PCP questions and specialist responses were in regions outside the Chaplain region, within the province of Ontario. It took a median of 1.11 days (IQR = 0.3–4.7) for specialists to respond to the cases, and 95% (*n* = 58) of cases received a response within 7 days of the submission, with a median of 1 question-response interaction. Specialists recorded a median of 15 min to respond to an eConsult (IQR = 10–20), leading to a median cost of 50.00 Canadian dollars (IQR = 33.33 – 66.66) per eConsult.

**Table 1:** eConsult utilisation data for cases related to frailty submitted in 2019

	**Percent (n) *N* = 61**	**Median**	**Mean**
Cases submitted by nurse practitioner vs physician	13 (8)	-	-
Specialist response time (days)	-	1.11	2.61
Cases responded to in 7 days or less	95 (58)	-	-
Specialist time billed per eConsult (minutes)	-	15.0	17.4
Cost per eConsult (Canadian dollars)	-	50.00	57.92
Cases with only 1 question/response interaction	83 (51)	-	-

The 61 eConsults analyzed were sent to 24 different medical specialty and subspecialty groups (Table 2). The three most common were cardiology (*n* = 7; 11%); gastroenterology (*n* = 7; 11%); and endocrinology (*n* = 6; 10%). Among the 61 cases, one case was submitted to each of three geriatric specialties offered through eConsult: geriatric medicine, GeriMedRisk-clinical pharmacology, and GeriMedRisk-psychiatry. A total of 97 questions were asked across the 61 eConsults. In 49% (*n* = 30) of cases, PCPs asked a single question, 43% (*n* = 26) included two questions, and 8% (*n* = 5) included three.

**Table 2:** eConsult specialty groups to which frailty-related cases were submitted (*N* = 61)

Specialty	Number of eConsults (%)
Cardiology	7 (11)
Gastroenterology	7 (11)
Endocrinology	6 (10)
Dermatology	5 (8)
Hematology	4 (7)
Endocrinology—Osteoporosis	4 (7)
Rheumatology	3 (5)
Pain Medicine	3 (5)
General Surgery	3 (5)
Vascular Surgery	2 (3)
Diabetes Education	2 (3)
Ophthalmology	2 (3)
Nephrology	2 (3)
GeriMedRisk—Clinical Pharmacology	1 (2)
Orthopaedics	1 (2)
ENT—Otolaryngology—Head and Neck Surgery	1 (2)
Palliative Care	1 (2)
Parkinsons Movement Disorders—Grimes	1 (2)
Psychiatry	1 (2)
Geriatric Medicine—Mind	1 (2)
Stroke_TIA	1 (2)
Urology	1 (2)
GeriMedRisk—Psychiatry	1 (2)
Wound Care	1 (2)

### Clinician question types and content

**Fig. 1:**
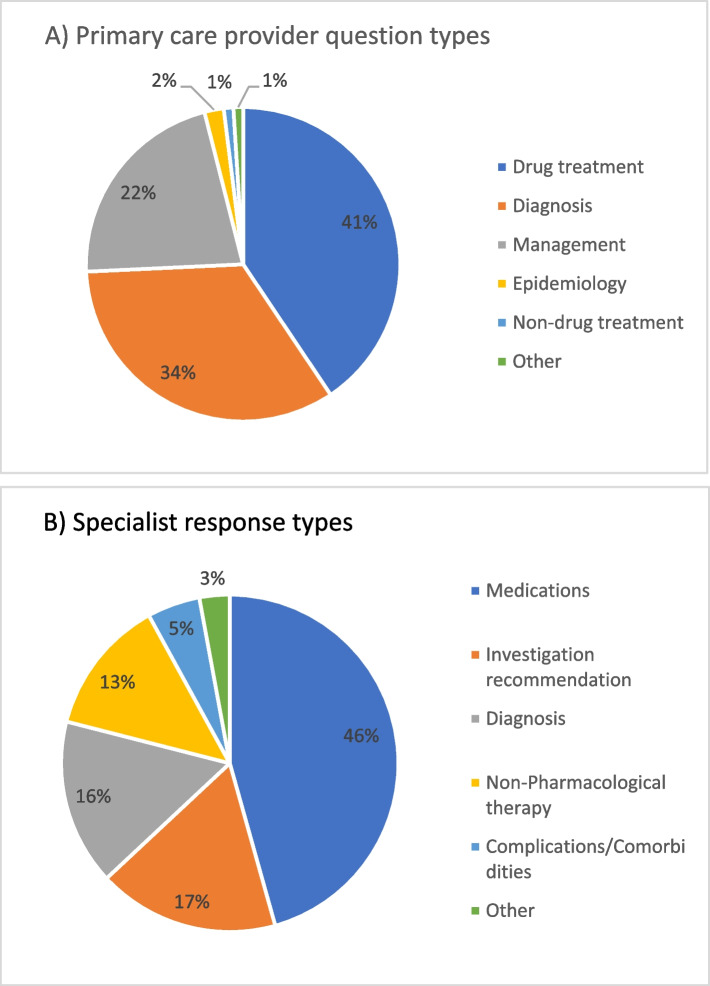
**A** Types of questions asked by primary care providers through eConsult about patients with frailty (*N* = 97); **B** Types of specialist responses to eConsults for patients with frailty (*N* = 138)

**Fig. 2:**
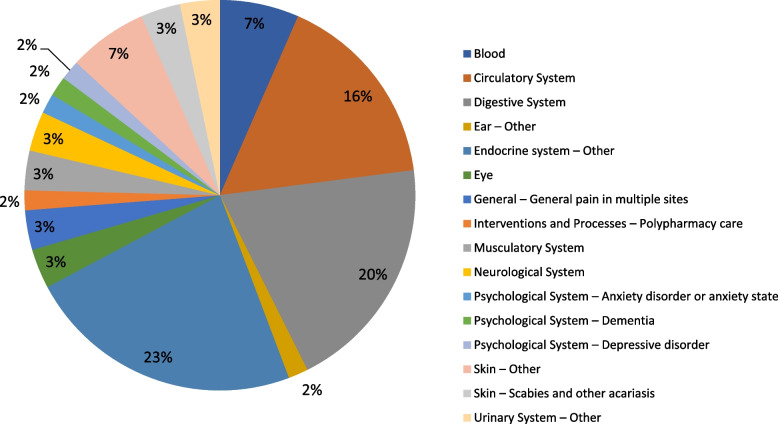
Content classification of primary care provider questions submitted through eConsult for patients with frailty

Due to low numbers, the classifications of question types and content have been collapsed across subcategories and are presented under the broader categories. Of the 97 questions asked across the 61 cases, the most common question types related to drug treatment (*n* = 40, 41%), management (*n* = 33, 34%), and diagnosis (*n* = 21, 22%) (See Fig. 1A). The clinical content of the 61 eConsult cases spanned thirteen areas, most commonly the endocrine (*n* = 14, 23%), digestive (*n* = 12, 20%) and circulatory (*n* = 10, 16%) systems, followed by skin (*n* = 6, 10%), blood (*n* = 4, 7%), and the psychological system, including dementia and depressive disorders (*n* = 3, 5%). The remaining 20% of cases (*n* = 12) were spread across 7 different content areas (See Fig. 2).

### Specialist responses

In 80% (*n* = 49) of the eConsults, specialists provided more than one answer, resulting in 138 answers by specialists across the 61 eConsults. Specialist answers most often pertained to medications (*n* = 63, 46%), recommendations for clinical investigation (*n* = 24, 17%), and diagnoses (*n* = 22, 16%) (See Fig. 1B). In 57% (*n* = 35) of the eConsults, specialists made no recommendation regarding a referral, whereas for 13% (*n* = 8) of cases a specialist visit was recommended, and for 20% (*n* = 12) they indicated the potential need for future referral. Referrals to allied health professionals and specialized geriatric assessments were given to 3% (*n* = 2) of cases, and referrals to community services were never made. In 20% (*n* = 12) of the eConsults, the specialist referenced specific guidelines or sources of information in their response.

### Impact of eConsult on care for patients living with frailty

**Fig. 3:**
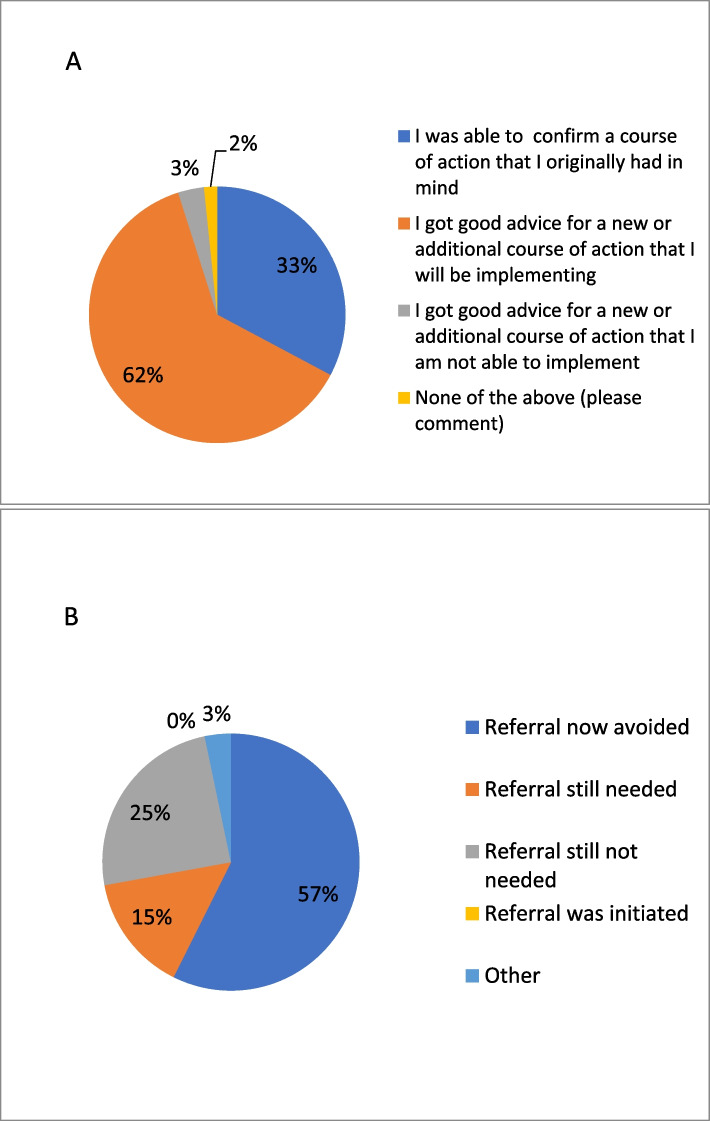
Primary care provider survey responses regarding the impact of eConsult: **A**) on the course of action in caring for their patient with frailty; **B**) on the need for a face-to-face referral with a specialist for their patient with frailty

According to the survey data provided by PCPs upon closing the eConsult cases, PCPs received advice for a new or additional course of action that was not originally contemplated in 62% (*n* = 38) of cases. In 57% (*n* = 35) of cases, PCPs had originally contemplated a referral, but did not refer after receiving advice through eConsult (See Fig. 3A). In 15% (*n* = 9) of cases a referral was still needed after the eConsult was completed. The eConsult process never resulted in a referral that wasn’t originally contemplated by the PCP (See Fig. 3B). In 95% (*n* = 58) of cases, PCPs rated the specialists’ responses as helpful or very helpful.

## Discussion

Though there are important potential benefits of detecting and managing frailty in primary care settings, including increased equitable access to care, reduction of healthcare costs, decreased hospital visits, and improved health outcomes [16], little is known about the approaches used and challenges faced by PCPs in providing care for older adults living with frailty [8, 11]. The current study of PCP questions through the eConsult system is one of the first to provide insight into PCPs’ knowledge gaps and approaches to care for this patient population. We identified 61 eConsult cases closed in 2019 in the ChamplainBASE™ eConsult service where PCPs described their over-65-year-old patient as “frail”. eConsult questions about these patients were most commonly about drug treatment and were addressed to a number of different disease- or organ-specific specialty groups, most commonly cardiology, and only seldom to geriatric medicine groups. eConsult proved to be a valuable tool for PCPs providing frailty care – specialists responded quickly, and referrals were avoided for 57% of the cases.

### Patient-centred care

Recognition of frailty in primary care is an opportunity for a holistic consideration of care needs of patients living with frailty [9]. However, PCP questions through the eConsult platform focused on episodic and specific body system issues or diseases. Content classification revealed that all 61 cases were about diseases or organ systems, and none were about social problems, functioning, or interventions and processes. By the same token, specialists consulted were in most cases organ- or disease-focused (See Table 2). While the eConsult ChamplainBASE™ services offer access to several geriatric specialty groups, only five percent of eConsults in this sample were sent to one of these groups. This finding is in line with a previous study of ChamplainBASE™ eConsult service use in long-term care which reported that eConsults directed to geriatric-specific specialities made up only 11% of those sent [13]. It is also worth noting that in response to the PCP questions, specialists rarely recommended referrals, but when they did, these referrals were mainly to specialists, rather than geriatric programs, allied health professionals, or community services. Ambagtsheer et al. have previously reported that PCPs could benefit from training and support in the provision of patient-centred frailty care [11]. This should include support in the use of available tools and programs to increase communication and access to specialist advice, such as geriatric-specific specialties on eConsult.

### Drug-related questions

The high proportion of drug-related questions (41%) in our dataset substantiates well-recognised challenges related to medication optimization in older adults with frailty [17]. Polypharmacy increases the risk of onset or exacerbation of frailty, hospital admissions, mortality, and healthcare expenditure, and these risks are higher for patients with frailty compared to those without [18]. Several approaches have been proposed to better manage prescriptions and limit adverse drug events among older adults. These include regular medication reviews, Patient-Centred Prescription Models, screening tools to identify potentially inappropriate medications [17, 19], and GeriMedRisk, a consultation service that provides interdisciplinary clinical recommendations to optimize medications for older patients [20]. However, evaluations of these approaches are still limited. Despite the high volume of drug-related questions we observed, the GeriMedRisk service available through eConsult was only accessed in one case within our sample. Given the frequency of drug-related eConsult questions and the potential impact of inappropriate prescriptions in patients living with frailty [17, 21], a more in-depth investigation into the nature of the drug-related eConsult questions and responses is warranted.

### Feasibility and impact of eConsult for this patient population

Despite the complexity involved in caring for patients living with frailty as evidenced by half of the eConsults involving more than one question and 80% including more than one response or recommendation from the specialist, specialist responses were submitted within a median of 1.1 days, specialists recorded a median of 15 min per case, and 57% of referrals initially contemplated by the PCP were avoided. These results suggest that eConsult is a feasible and valuable tool for improving care for complex older patients in primary care settings. Direct and indirect cost savings for the patient, their families and the health care system can be accrued by the reduction of in-person encounters with specialists. Less travel, less time spent by families and caregivers, and minimizing the burden of multiple healthcare visits for patients likely suffering from comorbidity and mobility limitations represent some of the key benefits.

While information and communication technologies (ICT) hold the promise of providing low-cost and high-efficiency methods for managing frailty more proactively, they remain understudied and underused [22, 23]. Among European Union countries, only a third report the use of ICT solutions to prevent or manage frailty, and only 15% allocate funding to support their implementation [22]. The need for more integrated care and better communication between care providers has been emphasized by older adults, their caregivers and health care providers as a key component in improving frailty care [24]. ICT such as eConsult can optimize communication and integration of care. To our knowledge, this is the first study to examine the use of eConsult for patients with frailty. Previous studies on the use of eConsult in the long-term care homes reported similar benefits, with 36% of referrals avoided [13, 25]. The role of eConsult in promoting patient-centred and holistic care for individuals living with frailty is an important area for future exploration.

### Limitations

This study has a few important limitations. First, our analysis was limited to a sample of 61 cases from the eastern region of Ontario, Canada, submitted to the eConsult platform in a single year. Replication in larger number of cases and in different regions may yield further insights into PCP questions and the use of eConsult for older adults living with frailty. Furthermore, we identified patients living with frailty through a search for the terms “frail” or “frailty” in the eConsult communication log. Patients living with frailty but not described by the PCP using the term “frail” are not captured in our sample. Alternate methods for identifying eConsult cases related to frailty may shed new light on PCP knowledge gaps and the value of eConsult for frailty care. Finally, since eConsults are PCP-initiated communications with specialists, they may not fully capture PCPs’ knowledge gaps and approaches to frailty care. Though our sample included both physicians and nurse practitioners, and those working in the community as well as in long-term care settings, it is worth noting that those who chose to use eConsult may not be representative of all primary care providers.

## Conclusion

By leveraging data available through eConsult, this study provides novel information on PCP knowledge gaps and approaches to care for patients living with frailty. In addition, it demonstrates how eConsult can be used to facilitate the delivery of care for older adults in the primary care setting by supporting communication between healthcare providers. Through this study, we have shown how PCPs often use the service to obtain drug-related and disease-specific information for patients identified as frail. Further investigation into tailoring specialty services offered through eConsult to the needs of patients living with frailty and training PCPs to access relevant services are warranted.

### Supplementary Information

**Additional file 1:** **Appendix A.** Taxonomy of Generic Clinical Questions (TGCQ). **Appendix B.** International Classification for Primary Care 3 (ICPC-3) – Categories considered for the classification of frailty-related eConsults. **Appendix C.** Close-out survey completed by primary care providers at the end of each eConsult.

## Data Availability

The datasets generated and/or analysed during the current study are not publicly available because they contain confidential patient identifiable information. Data that do not include patient identifiable information are available from the corresponding author on reasonable request.

## References

[1] Bergman H, Ferrucci L, Guralnik J, Hogan DB, Hummel S, Karunananthan S (2007). Frailty: an emerging research and clinical paradigm–issues and controversies. J Gerontol A Biol Sci Med Sci.

[2] Dent E, Martin FC, Bergman H, Woo J, Romero-Ortuno R, Walston JD (2019). Management of frailty: opportunities, challenges, and future directions. Lancet.

[3] Hoogendijk EO, Afilalo J, Ensrud KE, Kowal P, Onder G, Fried LP (2019). Frailty: implications for clinical practice and public health. Lancet.

[4] Jørgensen R, Brabrand M (2017). Screening of the frail patient in the emergency department: a systematic review. Eur J Intern Med.

[5] Sinha S, McKee A, Dunning J, Wong I, Nicin M, Muscedere J. We Can’t Address What We Don’t Measure Consistently: Building Consensus on Frailty in Canada. National Institute on Ageing at Ryerson University, Ryerson University, Toronto; 2018. Available from: https://static1.squarespace.com/static/5c2fa7b03917eed9b5a436d8/t/5dd3f6b8dcacb37f075231e9/1574172344714/Frailty%2BPaper%2BLayout_updated%2BCover.pd+-+Copy.pdf.

[6] Reeves D, Pye S, Ashcroft DM, Clegg A, Kontopantelis E, Blakeman T (2018). The challenge of ageing populations and patient frailty: can primary care adapt?. BMJ.

[7] Vellas B, Cestac P, Moley JE (2012). Implementing frailty into clinical practice: we cannot wait. J Nutr Health Aging.

[8] Abyad A (2021). Is primary health care capable of addressing frailty?. Eur Geriatr Med.

[9] Lawless MT, Archibald MM, Ambagtsheer RC, Kitson AL (2020). Factors influencing communication about frailty in primary care: A scoping review. Patient Educ Couns.

[10] Frost R, Robinson K, Gordon A, Caldeira de Melo R, Villas Boas PJF, Azevedo PS (2023). Identifying and Managing frailty: a survey of UK healthcare professionals. J Appl Gerontol.

[11] Ambagtsheer RC, Beilby JJ, Visvanathan R, Dent E, Yu S, Braunack-Mayer AJ (2019). Should we screen for frailty in primary care settings? A fresh perspective on the frailty evidence base: a narrative review. Prev Med.

[12] Liddy C, Maranger J, Afkham A, Keely E (2013). Ten Steps to Establishing an e-consultation service to improve access to specialist care. Telemedicine e-Health.

[13] Helmer-Smith M, Fung C, Afkham A, Crowe L, Gazarin M, Keely E (2020). The feasibility of using electronic consultation in long-term care homes. J Am Med Dir Assoc.

[14] Ely JW, Osheroff JA, Gorman PN, Ebell MH, Chambliss ML, Pifer EA (2000). A taxonomy of generic clinical questions: classification study. BMJ.

[15] World Organization of Family Doctors. ICPC-3: International Classification of Primary Care – 3rd Revision. 2020. Available from: https://www.icpc-3.info/. Cited 2021 Mar 22.

[16] Abbasi M, Rolfson D, Khera AS, Dabravolskaj J, Dent E, Xia L (2018). Identification and management of frailty in the primary care setting. CMAJ.

[17] Molist-Brunet N, Sevilla-Sánchez D, Puigoriol-Juvanteny E, Barneto-Soto M, González-Bueno J, Espaulella-Panicot J (2022). Improving individualized prescription in patients with multimorbidity through medication review. BMC Geriatr.

[18] Poudel A, Peel NM, Nissen LM, Mitchell CA, Gray LC, Hubbard RE (2016). Adverse outcomes in relation to polypharmacy in robust and frail older hospital patients. J Am Med Dir Assoc.

[19] Scott IA, Hilmer SN, Reeve E, Potter K, Le Couteur D, Rigby D (2015). Reducing inappropriate polypharmacy: the process of deprescribing. JAMA Intern Med.

[20] Ho JMW, Tung J, Maitland J, Mangin D, Thabane L, Pavlin JM (2018). GeriMedRisk, a telemedicine geriatric pharmacology consultation service to address adverse drug events in long-term care: a stepped-wedge cluster randomized feasibility trial protocol (ISRCTN17219647). Pilot Feasibility Stud.

[21] Counter D, Millar JWT, McLay JS (2018). Hospital readmissions, mortality and potentially inappropriate prescribing: a retrospective study of older adults discharged from hospital. Br J Clin Pharmacol.

[22] Cruz AM, Monsalve L, Ladurner AM, Jaime LF, Wang D, Quiroga DA (2021). Information and communication technologies for managing frailty: a systematic literature review. Aging Dis.

[23] Selak Š, Bacaicoa OA, Gabrovec B (2019). Can we manage frailty at individual level by the use of information and communication technologies: a narrative literature review. Slovenian Med J.

[24] Shaw RL, Gwyther H, Holland C, Bujnowska-Fedak M, Kurpas D, Cano A (2018). Understanding frailty: meanings and beliefs about screening and prevention across key stakeholder groups in Europe. Ageing Soc.

[25] Fung C, Shah S, Helmer-Smith M, Levi C, Keely E, Liddy C. What are the clinical questions asked by primary care providers in long-term care through eConsult? A retrospective study of eConsult cases. [Manuscript in progress]. 2021. 10.1177/23337214211032055 PMC8404619 34471649

