# Supplementary material for: What do primary care providers want to know when caring for patients living with frailty? An analysis of eConsult communications between primary care providers and specialists
Source: BMC Health Serv Res. 2024 Jan 16;24:76. doi: 10.1186/s12913-024-10542-x (PMC10790473; doi:10.1186/s12913-024-10542-x)
Supplement: Supplementary file 1 — Additional file 1: Appendix A. Taxonomy of Generic Clinical Questions (TGCQ). Appendix B. International Classification for Primary Care 3 (ICPC-3) – Categories considered for the classification of frailty-related eConsults. Appendix C. Close-out survey completed by primary care providers at the end of each eConsult. [file 12913_2024_10542_MOESM1_ESM.docx]

**Appendix A:** Taxonomy of Generic Clinical Questions (TGCQ)

Diagnosis – Criteria/manifestations

Diagnosis – Determining most appropriate test (lab, ECG, imaging, biopsy, skin test, element of physical exam, etc.)

Diagnosis – interpretation of test results/clinical finding

Diagnosis – Other

Drug Treatment – How/when to prescribe a drug

Drug Treatment – How/when to deprescribe a drug

Drug Treatment – Safety/adverse effects/Interactions

Drug Treatment – Other

Non-Drug Treatment – Determining most appropriate treatment or procedure (non-drug ± drug)

Non-Drug Treatment – When/how to use a non-drug treatment or procedure

Non-Drug Treatment – Other

Management – How to manage (not specifying diagnostic or therapeutic)

Management – Questions about referrals to other providers for management (not for diagnosis or treatment)

Management – Questions about referrals to community services

Management – doctor – patient/family communication (providing advice, discussing difficult situation, compliance)

Management – Other

Epidemiology – Etiology

Epidemiology – Course/prognosis

Epidemiology – Other

Education – Clinical training or information needs

Education – Patient education resources

Education – Other

Nonclinical – Administration/ethics/legal/other

Nonclinical – Other

Not elsewhere classified

**Appendix B:**

International Classification for Primary Care 3 (ICPC-3) – Categories considered for the classification of frailty-related eConsults

General – General pain in multiple sites

General – General weakness or tiredness

General – Fall of unknown origin

General – Other specified abnormal result investigation

General – Concern about or fear of medical treatment

General – Other specified and unknown infectious diseases

General – Multiple trauma and injuries

General – Poisoning by medical agent

General – Adverse effect of medical agent

General – Complication of medical treatment

General – Other

Blood

Digestive System

Eye

Genital System

Ear – Presbycusis

Ear – Deafness

Ear – Other

Circulatory System

Musculatory System

Neurological System

Psychological System – Chronic alcohol problem

Psychological System – Medication abuse

Psychological System – Memory or attention problem

Psychological System – Dependence on others

Psychological System – Dementia

Psychological System – Delirium

Psychological System – Affective psychosis

Psychological System – Anxiety disorder or anxiety state

Psychological System –Depressive disorder

Psychological System– Other

Respirology System – Aspiration pneumonia

Respirology System – Other

Skin – Herpes zoster

Skin – Onychomycosis

Skin – Candidiasis skin

Skin – Scabies and other acariasis

Skin – Head lice

Skin – Laceration or cut

Skin – Other

Endocrine system – Loss of appetite

Endocrine system – Weight loss

Endocrine system – Other

Urinary System – Urinary frequency or urgency

Urinary System – Urine incontinence

Urinary System – Urinary retention

Urinary System – Other

Social Problems – Problems associated with finances

Social Problems – Legal problem

Social Problems – Social welfare problem

Social Problems – Person awaiting admission to elderly/nursing home

Social Problems – Other

Interventions and Processes – Specific physical function test

Interventions and Processes – Standard mental, cognitive, physical functioning tests and questionnaires

Interventions and Processes – Dementia (management) programme

Interventions and Processes – Depression (management) programme

Interventions and Processes – Polypharmacy care

Interventions and Processes – Complex and integral care programme

Interventions and Processes – Frailty elderly programme

Interventions and Processes – Palliative care and end of life care

Interventions and Processes – Formulation of plan care, management, treatment or intervention

Interventions and Processes – Other

Functioning (related) – Focusing attention

Functioning (related) – Thinking

Functioning (related) – Solving problems

Functioning (related) – Making decisions

Functioning (related) – Undertaking a single task

Functioning (related) – Carrying out daily routine

Functioning (related) – Communicating with – receiving – spoken messages

Functioning (related) – Speaking

Functioning (related) – Using communication devices and techniques

Functioning (related) – Walking long distances and short distances

Functioning (related) – Climbing (steps)

Functioning (related) – Moving around within the home

Functioning (related) – Moving around outside the home and other buildings

Functioning (related) – Moving around using equipment

Functioning (related) – Using transportation

Functioning (related) – Driving

Functioning (related) – Washing oneself

Functioning (related) – Caring for body parts

Functioning (related) – Toileting

Functioning (related) – Dressing

Functioning (related) – Eating

Functioning (related) – Drinking

Functioning (related) – Looking after one’s health

Functioning (related) – Doing housework

Functioning (related) – Energy level

Functioning (related) – Sleep functions

Functioning (related) – Attention functions

Functioning (related) – Memory functions

Functioning (related) – Emotional functions

Functioning (related) – Seeing functions

Functioning (related) – Heating functions

Functioning (related) – Balance

Functioning (related) – Dizziness

Functioning (related) – Exercise tolerance functions

Functioning (related) – Muscle power functions

Functioning (related) – Drug (medication)

Functioning (related) – Other

**Appendix C:** Close-out survey completed by primary care providers at the end of each eConsult

**Q1. Which of the following best describes the outcome of this eConsult for your patient?**

1. I was able to confirm a course of action that I originally had in mind
2. I got good advice for a new or additional course of action that I will be implementing
3. I got good advice for a new or additional course of action that I am not able to implement
4. None of the above (please comment)

**Q2: As a result of this eConsult would you say that:**

1. Referral was originally contemplated but now avoided at this stage
2. Referral was originally contemplated and is still needed
3. Referral was not originally contemplated and is still not needed
4. Referral was not originally contemplated, but eConsult process resulted in a referral being initiated
5. Other (please explain)

**Q3: How helpful and/or educational was this response in guiding your ongoing evaluation or management of the patient?**

Minimal 1 2 3 4 5 Very Valuable.

**Q4: This eConsult addresses an important clinical problem that should be incorporated into upcoming CME events**

Strongly Disagree 1 2 3 4 5 Strongly Agree.

**Q5: We would value any additional feedback you provide [Comments for the specialist will be forwarded to her/him]:**

(Comment Box)
